# ENIGMA-TRS phase 3 trial: designing and implementing a global study of evenamide in treatment-resistant schizophrenia

**DOI:** 10.1192/j.eurpsy.2026.12199

**Published:** 2026-07-31

**Authors:** R. Anand, A. Turolla, G. Chinellato, F. Sansi, R. Hartman

**Affiliations:** 1 APC, St.Moritz, Switzerland; 2 Newron Pharmaceuticals SpA, Bresso, Italy; 3 NeurWrite, Morristown, United States

## Abstract

**Introduction:**

Treatment-resistant schizophrenia (TRS) is associated with high risk for morbidity, mortality and hospitalization, as available antipsychotics (APs) are ineffective in treating these patients (Siskind et al, *BrJPsychiatry* 2022). Clozapine is the only approved treatment for TRS worldwide, but its usage is limited to 5% in US and 15% in EU (Warnez et al, *BMC Psychiatry* 2014) due to safety concerns and blood monitoring requirements. Emerging evidence implicates excessive glutamatergic rather than dopaminergic activity in patients with TRS (Demjaha et al, *BiolPsychiatry* 2014), explaining the poor response to first- and second-generation APs (FGA/SGA) and pointing to the need for novel drugs targeting the glutamate system.

Evenamide has proved to reduce aberrant hippocampal glutamatergic activity and hyperdopaminergic firing. Clinical benefits of evenamide as an add-on to SGA were observed in a long-term uncontrolled study in TRS patients (Anand et al, *IJNP* 2025) and replicated in a short-term placebo-controlled study in inadequate responders (Anand et al, *Neuropharm* 2025). ENIGMA-TRS 1 (EveNamIde’s Glutamate Modulation Ameliorates TRS) is a phase 3, international, 1-year, randomized, double-blind, placebo-controlled trial with the primary efficacy endpoint at 12 weeks and long-term efficacy endpoints at 26 and 52 weeks. It evaluates efficacy, tolerability and safety of 15 and 30 mg BID of evenamide as add-on in TRS patients on SGA (including clozapine).

**Objectives:**

To present considerations and challenges faced in the design of ENIGMA-TRS 1 trial.

**Methods:**

The study is conducted at >80 centers in 20 countries across Europe, Asia, Latin and North America. This implies high heterogeneity in healthcare systems, languages, evaluation of patients’ illness through subjective measures (PANSS/CGI). Moreover, it requires approval from numerous regulatory authorities. Other challenges relate to the patient population, e.g. adoption of appropriate criteria for TRS diagnosis, assessment of compliance with current AP, need for caregiver.

**Results:**

For the design of the study, multiple discussions with regulatory authorities and advisory boards with international experts were conducted to ensure regulatory requirements are fulfilled. A rater training program has been developed and implemented to standardize rating practices across centers. Patients with TRS are selected according to TRRIP consensus criteria, severity of illness is critically evaluated, and an independent committee ensures high standards in enrollment. Compliance with current AP is assessed through plasma level blood monitoring to confirm that patients are truly resistant rather than noncompliant.

**Conclusions:**

Despite many challenges, several strategies have been undertaken to guarantee global high standards by relying on internationally accepted criteria, ensure homogeneity in patients’ evaluation, and provide reliable data.

**Disclosure of Interest:**

R. Anand Consultant of: Newron Pharmaceuticals SpA, A. Turolla Employee of: Newron Pharmaceuticals SpA, G. Chinellato Employee of: Newron Pharmaceuticals SpA, F. Sansi Employee of: Newron Pharmaceuticals SpA, R. Hartman Consultant of: Newron Pharmaceuticals SpA

